# The impact of digital logistics under the big environment of economy

**DOI:** 10.1371/journal.pone.0283613

**Published:** 2023-04-06

**Authors:** Liuhua Zhang, Tianbao Gong, Yanan Tong

**Affiliations:** 1 Qiannan Normal University for Nationalities, Duyun, Guizhou, China; 2 Shenzhen Tiansu Calibration and Testing Co. Ltd., Longgang District, Shenzhen, Guangdong, China; 3 Guangzhou Kumu Brand Design Co. Ltd, Haizhu District, Guangzhou, Guangdong, China; Balochistan University of Information Technology Engineering and Management Sciences, PAKISTAN

## Abstract

Digital logistics techniques are important for business applications that contribute to economic growth. The modern supply chain or logistics seeks to implement a large-scale smart infrastructure incorporating data, physical objects, information, products, and business progressions. The business applications are utilized various intelligent techniques to maximize the logistic process. However, the logistic process suffers due to transportation costs, quality, and multinational transportation. These factors frequently affect the region’s economic growth. In addition, most cities are located in remote areas that receive improper logistic support, which minimizes business growth. So, this work analyzes the impact of digital logistics on the region’s economy. The Yangtze River economic belt region, which includes almost 11 cities, is chosen for analysis. The gathered information is processed by Dynamic Stochastic Equilibrium with Statistical Analysis Modelling (DSE-SAM), which predicts the correlation and influence of digital logistics on economic development. Here, the judgment matrix is constructed to reduce the difficulties of data standardization and normalization processes. Then entropy model and statistical correlation analysis are utilized to improve the overall impact analysis process. Finally, the developed DSE-SAM based created system efficiency is compared with the other economic models, such as Spatial Durbin Model (SDM), Coupling Coordination Degree Model (CCDM), and Collaborative Degree Model (CDM). The results of the suggested DSE-SAM model achieve a high correlation of urbanization, logistics, and ecology in the Yangtze River economic belt region compared to other regions.

## 1. Introduction

The automatic and digitization process of goods movement is named "Digital Logistics," [1] which helps to minimize manual efforts in various business applications. Almost every business process utilizes Artificial Intelligence (AI), the Internet of Things (IoT), Machine Learning (ML), and Blockchain techniques [2] to enhance the digital logistic process in supply chain management. The digital logistic process impacts various parts of the supply chain, including supply chain forecasting analysis, customer notification, inventory management, transport management, and warehouse management [3, 4]. Compared to traditional logistics, digital logistics has several benefits, such as real-time flexibility, streamlined inventory management, task automation, and continuous improvement. The digitized logistics process gives various operations and maximizes the customer experience with minimum shipping and cost [5]. However, while integrating the products, the digitally transformed logistic process needs to manage the data quality, provide excellent customer support, and maximize the user experience. These logistic difficulties, such as transportation, cost, and availability of resources, impact the economy because companies are ready to provide flexibility after changing the inventory control [6]. Logistics is the pillar of the national economy; it also gives the platform to create the connection between the production and consumption of the rural and urban areas. In addition, digital logistics eliminate the difficulties between social production linking and region-resident consumption. The technological development in industries and the logistic sector create an impact on economic growth [7]. Therefore, enterprises and the government have made a strenuous effort in digital logistics, according to the China E-state council [8]. In this council, they released two documents, such as e-efficient logistics and made in China 2025, which focus on improving their country’s economic growth. Hence, the China government is slowly moving to the intelligent logistics industry and creating various logistics companies such as Shunfeng, Cainiao, Jingdong, and Meituan in China [9]. The developed smart enterprises are planning to maximize their competitiveness and efficiency.

The digitized logistic [10] process has a set of supply groups, demand groups, and medical operators with operation centers. These groups help improve industrial performance and efficiency by providing a complete and large infrastructure platform. The development of technologies and social demand requires large-scale e-logistics to maximize business growth and the economy [11]. In addition, transportation maximizes the logistics enterprise’s income and enhances the production flow between the provinces. The production cost is minimized by transporting the goods from the central regions, which causes maximizing economic growth [12]. Factors such as logistic platforms, infrastructure construction, education, cross-regional culture, science, and technology influence the logistic-based economy. Therefore, the state council formulated a Medium-long term plan for smart logistic enterprises to manage the sustainability of logistics [13]. Logistic sustainability will be achieved with the help of convenience, advanced technology, reasonable layout, environmental protections, efficiency, and safety by 2020. In Guangdong province, the Pearl River Delta Economic region in China has 14 cities in which various smart logistic industries are constructed to improve economic growth [14, 15]. This Guangdong province has 85% of the Gross Domestic Product (GDP) and 70% of the population, which are more relatable to the e-logistics. The Pearl River region handled various situations and difficulties during goods transportation [16]. Therefore, Guangdong province concentrates on smart logistics to reduce manual problems and maximize economic status. At the time, China government focuses on the cross-regional section to strengthen the multi-model transportation system [17] and logistic networks to improve the logistics industry’s performance. Moreover, this process manages the logistics industry’s health, sustainability, and overall business efficiency.

In China, Guangdong province [18], the Pan-Pearl delta region has several foreign trade locations and gateway; hence, this region has contributed to economic aggregation for the last 30 consecutive years. According to the various analysis, 20% of economic output is obtained from the Guangdong-Hong western area such as Maoming, Yangjiang, and Zhanjiang; 80% of economic output attains in Macao Greater Bay areas like Dongguan, Foshan, Shenzhen, Guangzhou, Huizhou, Zhuhai, Jiangmen, and Zhaoqing [19]. This discussion shows that the Pearl River region has an imbalanced economic zone because of the developed logistics, partially convenient transportation, and frequent overseas trade. Along with this, northern mountain transportation is more difficult because of the inconvenient area, which causes complexity in good transport. Therefore, several studies concentrate on the influential logistic industries to improve the country’s economic growth. The logistic industries require development to enhance their trade and economy by exchanging goods. However, it is difficult for most industries to justify how the logistic enterprises maximize the regional economy and how it spills over the region’s economy. According to the discussion, this research study analyzes a few logistic industry models to analyze the region’s economic growth. In addition, how the logistic process maximizes the country’s growth and respective economic model is discussed. Modern macroeconomic theory is utilized in dynamic stochastic general equilibrium (DSGE) models to perform policy analysis and to explain and forecast the comovements of aggregate time series over the business cycle. To that end, the model separates the effects of inflation, GDP growth, and the short-term interest rate. Statistical modeling involves using statistical methods to build a representation of data, analyze it for patterns, and draw conclusions. Statistics in economics focuses on the gathering, processing, and interpreting of data relevant to economics. It’s a useful tool for analyzing economic theories and drawing connections between supply, demand, pricing, production, etc. Based on the survey, there are several issues with existing methods in achieving high economic growth and predicting the impact of digital logistics. Hence, this paper uses Dynamic Stochastic Equilibrium with Statistical Analysis Modelling (DSE-SAM) to analyze digital logistics’ impact on economic growth.

Then the main contribution of this study is listed as follows.

- To improve the correlation analysis process on the logistic industry and region’s economic growth by applying the Dynamic Stochastic Equilibrium with Statistical Analysis Modelling (DSE-SAM)
- To manage the primary and secondary indicators, unique index values are computed to minimize the computation difficulties.
- To reduce the difficulties in the remote location areas related to economic growth by analyzing the correlation between the indicators.

The above research objective is achieved by applying the Dynamic Stochastic Equilibrium with Statistical Analysis Modelling (DSE-SAM) approach. This approach analyzes the economic policy, strategies, and methods to investigate the logistic impacts. In addition, the statistical analysis modeling helps to explore each indicator that identifies the logistic impacts on economic growth.

This manuscript is formulated as follows: Section 2 analyzes the various economic model to analyze the logistic industry relationship between economic growth. Section 3 discusses the methods and materials, section 4 evaluates the system’s efficiency, and the conclusion is described in section 5.

## 2. Link between the logistic industry and economic growth

The regional economy and logistics industry have mutually promoted and are interdependent with each other. In downstream and upstream industries, supply chain logistics played a crucial role in improving the country’s economic growth. The successful creation of logistic industries leads to maximizing economic growth and provides the energy to highlight the transformation between the regions. In addition, this logistic industry supports regional competitiveness, adjusting the economic structure, economic growth, and integration construction. The relationship between logistics and economic development is analyzed by using various models. Here, a few models are discussed to understand the impact on economic growth.

### 2.1 Coupling coordination degree model

This section discusses Ye et al., 2022 [20] utilized the Coupling Coordination Degree Model (CCDM) to analyze the logistic industries to develop the new ecological environment in the Yangtze River belt. This study utilizes the 11 provinces in the Yangtze River belt region collected from 2009 to 2018. Initially, the entropy approach processes the gathered details, identifying the weight value of loading information. This process generates a standardization matrix by analyzing the industry’s logistic information. Then, considering the industry having the n evaluation objects and m as the evaluation indicators, matrix construction is performed for positive and inversive objects.


$$Y_{ij}=\frac{X_{ij}-min(X_{j})}{\max\left(X_{j}\right)-\min\left(X_{j}\right)}$$ (1)

Eq (1) is applied to the positive type of objects; here *X*_*j*_ is denoted that the original data and the normalized output *Y*_*ij*_ is obtained from the minimum and maximum values of *X*_*j*_. If the indicators have a negative impact, then Eq (2) is utilized to compute the matrix.


$$Y_{ij}=\frac{\max\left(X_{j}\right)-X_{ij}}{\max\left(X_{j}\right)-\min\left(X_{j}\right)}$$ (2)

After normalizing the input values, the entropy value is obtained using Eq (3)

$$E_{j}=-\frac{1}{\ln\left(n\right)}\sum_{i=1}^{n}p_{ij}\;\ln\;p_{ij}$$ (3)

In Eq (3), *p*_*ij*_ is computed from the normalized output value *Y*_*ij*_ which means; pij=Yij∑i=1nYij. Here, *E*_*j*_ is denoted as the j^th^ index entropy value and computed for evaluation objects n. After that, the weight value is computed for every indicator that is performed according to the coefficient variability (Eq 4)

$$g_{i}=1-E_{j}$$ (4)

According to the coefficient variability, *g*_*i*_, weight *W*_*j*_ is estimated using Eq (5)

Wj=gi∑j=1mgi (5)

In Eq (5), the number of indicators represented as m, j^th^ index indicator weight values are denoted as *W*_*j*_. Along with this, the comprehensive expansion index *U*_*i*_ is computed using the normalized output *Y*_*ij*_ and weight value *W*_*j*_ that is defined in Eq (6)

$$U_{i}=\sum_{j=1}^{m}W_{j}Y_{ij}$$ (6)

Considering the above computation, the consistency between the elements in the development process is computed. Let’s assume the logistic industries have the three comprehensive expansion indexes, such as *U*_1_, *U*_2_ and *U*_3_ then the ecological environment and urbanization logistic correlation value is computed using Eq (7)

$$C=\frac{3*{\left(U_{1}*U_{2}*U_{3}\right)}^{1/3}}{U_{1}+U_{2}+U_{3}}$$ (7)

In Eq (7), the C value is used to determine the correlation level of economic growth and the respective logistics industry value. Here, C has values as (0,1); if C has 0 value, there is nothing relationship between the system; else, the system has a high correlation value. The computed C value helps to identify the relationship of inter-system interaction; hence it has been defined using Eq (8)

$$D={\left(C*T\right)}^{1/2}$$ (8)

In Eq (8), T is defined as the reconciliation coefficient, which is computed as *T* = *aU*_1_+*bU*_2_+*cU*_3_, *a*, *b and c* is represented as the pending coefficient; *a*+*b*+*c* = 1. D signified as the coupling coordination degree. After that, spatial distribution and relationship between elements are analyzed using exploratory spatial data analysis. Here, local and global spatial correlation values are investigated. According to the correlation value, the logistics industry’s positive and negative impact on economic growth is predicted effectively. Further, the research study is extended, and the Spatial Durbin Model (SDM) was recommended by Wei et al., 2022 [21]. The detailed working process of SDM and the correlation between the logistic enterprises and the region’s economic growth is described in the below subsection.

### 2.2 Spatial Durbin Model (SDM)

As discussed in section 1, only 20% of economic activities happen in the Yangtze River region. Therefore, industrial aggregation should be performed in this region to increase economic activity. This work uses the two hypotheses to analyze the strong relationship between logistics and economic development. ***Hypothesis 1*** covers the impact of the transport or logistic integration on industrial aggregation, and ***hypothesis 2*** transport integration level to improve the local industrial aggregation level and maximize the positive impact on their urban industry economic growth. The network structure considers these two hypotheses, and the urban aggregation transportation integration index value is computed. Initially, the degree centrality is calculated in which in-degree and out-degree centrality are computed. The centrality is computed using the *CD*(*i*) = *ki*; here, *ki* is defined as the city degree value (number of cities in the region). If the region has a high value of *ki* then the city region has a direct connection to the center. Then eigenvector centrality is computed with the help of *ki* which is defined in Eq (9)

$${CE}_{i}=ki=c\sum_{j=1}^{g}a_{ij}k_{j}$$ (9)

Along with this, betweenness centrality *CB*_*i*_ is calculated to identify the link between transportation and economic activities.


$${CB}_{i}=\sum_{j=1}^{N}\sum_{k=1}^{j-1}\frac{\varphi_{jk}(i)}{\varphi_{jk}};j\neq k\neq i,j<k$$ (10)

In Eq (10), the shortest path between the cities is denoted as *φ*_*jk*_, number of the minimum path in the cities are represented as *φ*_*jk*_(*i*) and the relevancy between the cities is mentioned as $\frac{\varphi_{jk}(i)}{\varphi_{jk}}$. Then the closeness centrality *CC*_*i*_ is computed with the help of cities (i, j) shortest path length that is defined as ${CC}_{i}=\sum_{j=1}^{N}1/d_{ij}$. Then, the average travel time is computed using Eq (11)

$${AT}_{i}=\sum_{j=1}^{n}\frac{\left(T_{ij}*M_{j}\right)}{M_{j}}$$ (11)

In Eq (11), cities (i, j) shortest travel time is defined as *T*_*ij*_, city j’s economic quality is mentioned as *M*_*j*_. The economic quality is computed as $\sqrt{City\;CDP*Population}$. The economic potential (*P*_*j*_) is computed from the time *T*_*ij*_ and economic quality *M*_*j*_; $P_{j}=\sum_{j=1}^{n}\frac{M_{j}}{T_{ij}}$. Additionally, Chinese cities’ statistical yearbooks and provinces are analyzed to get the highway freight volume and passenger capacity. After analyzing these indicators in the economic development region, the entropy weight technique is applied to compute the transportation integration index value in urban aggregation. Initially, the calculated indicators are de-dimensionalized, normalizing the data according to Eq (1). The weight values are assigned to network structures, and the traffic integration index value is computed according to the equalization procedure. Here, 0.5 is the weight value for the traffic network function and structure index. According to the above indicators, SDM is constructed for hypothesis 1 using Eq (12)

$$Y_{it}=\rho_{1}WY_{it}+\rho_{2}WX_{it}+\beta_{1}X_{it}+\varepsilon_{it}$$ (12)

In Eq (12), the spatial weight matrix is mentioned as W, autocorrelation between the dependent variables is represented as *WY*, independent variables autocorrelation is denoted as WX, the dependent variable spatial lag coefficient is *ρ*_1_ and independent variable spatial spillover denoted as *ρ*_2_. The spatial weight matrix *w*_*ij*_ is estimated using Eq (13)

$$w_{ij}=\left\{ \begin{array}{l} \;w_{ij}=1\;i\;is\;adjacent\;to\;j\\ w_{ij}=0\;i\;not\;adjacent\;to\;j\;or\;i=j \end{array}\right.$$ (13)

After computing the *w*_*ij*_, the industrial location entropy (IE) is estimated as ${IE}_{it}=\frac{q_{it}/Q_{it}}{q_{t}/Q_{t}}$; here, *q*_*it*_ is defined as a city i industrial GDP, the city i and t year GDP value is denoted *Q*_*it*_. For t year, the national industrial GDP value is denoted as *q*_*t*_ and *Q*_*t*_ is the national GDP value of t year. Then the industrial entropy for the hypothesis is formulated using Eq (14)

$${IE}_{it}=\lambda \sum_{j=1}^{n}W_{ij}{IE}_{jt}+\beta_{1}TI_{it}+\beta_{2}X_{it}+\theta_{1}\sum_{j=1}^{n}W_{ij}TI_{jt}+\theta_{2}\sum_{j=1}^{n}W_{ij}X_{jt}+\varepsilon_{it}$$ (14)

In addition to this, variables are analyzed to compute the labor cost in the transportation aggregation integration is computed which is evaluated using hypothesis 2, and it is formulated using Eq (15)

$${IE}_{it}=\lambda \sum_{j=1}^{n}W_{ij}{IE}_{jt}+\beta_{1}Wa_{it}+\beta_{2}{Ur}_{it}{+\beta }_{3}{Ex}_{it}+\theta_{1}\sum_{j=1}^{n}W_{ij}Wa_{jt}+\theta_{2}\sum_{j=1}^{n}W_{ij}{Ur}_{it}+\theta_{3}\sum_{j=1}^{n}W_{ij}{Ex}_{it}+\varepsilon_{it}$$ (15)

In Eq (15), the shortest travel time is computed while computing the *w*_*ij*_. According to the above SDM process, the shortest logistic transportation is identified between the cities. Here, constructed matrix and entropy values are highly incorporated to improve the province’s product development and economic growth. This work addresses the difficulties in urban aggregation-related product development and gives the practices to improve the economy based on logistic transportation. However, the research study requires further statistical analysis while exploring heterogeneity-related industrial information.

### 2.3 Collaborative Degree Model (CDM)

The following economic model is the Collaborative Degree Model (CDM), introduced by Guo et al., 2022 [22]. This work intends to analyze the connection between digital logistics and the economy in the Yangtze River belt region. During the analysis, data-driven measurement and procedure are applied to explore the coordination development between the logistic industry and the digital economy. The standardized data has been analyzed continuously, and the evaluation index is built to reflect the importance of logistics in the digital economy. The collaboration degree model is also applied to the Yangtze River belt region data, which is processed according to the collaboration policies degree and growth trend. This study uses the Anhui province, and the data is collected from 2013 to 2020; the gathered indicators are explored by using statistical measures. The collected data was analyzed using the data-standardization method, such as z-score and the min-max standardization process. The indicator maximum and minimum values are utilized to perform the standardization process. The hybrid system is created with the help of the coordination degree model, which utilizes the digital economy and digital logistic process. These two industries are operated in the progressive layers in which the order degree and collaborative degree model are computed in the hybrid system. The hybrid system has the conceptualization S1 and S2 in which S1 is utilized the digital economy, and S2 is the logistic system. Each conceptualization has a set of variables like order-parameter *e*_*ij*_ which has the limited between two parameters, such as *α*_*ij*_ and *β*_*ij*_. Then the order parameter variable’s positive effect-related degree value is computed using Eq (16).


$$U_{j}(e_{ji})=\frac{\left(e_{ji}-\alpha_{ji}\right)}{\left(\beta_{ji}-\alpha_{ji}\right)}\;i\in (1,k)$$ (16)

In addition, the negative impact of the order parameter degree value is calculated using Eq (16)

$$U_{j}(e_{ji})=\frac{\left(\beta_{ji}-e_{ji}\right)}{\left(\beta_{ji}-\alpha_{ji}\right)}\;i\in (k+1,n)$$ (17)

The above Eq (16 and 17) is utilized to determine the contribution of digital logistics to the logistics economy. If the computed *U*_*j*_(*e*_*ji*_) value closely related to 1 has a high correlation, and 0 means less correlation. Then subsystem order model is estimated by integrating the component order parameters. The integration process uses the average geometric method to estimate the subsystem model order, which is defined in Eq (18)

$$U_{j}(e_{ji})=\sqrt[{n}]{\left|\prod_{i}^{n}U_{j}(e_{ji})\right|}$$ (18)

The computed magnitude *U*_*j*_(*e*_*ji*_) degree value indicates that the system has a high contribution to the digital economy, and the order of the subsystems are computed using Eq (19 and 20)

$$U_{1}(s_{1})=\sqrt[{n}]{\left|\prod_{i}^{n}U_{1}(s_{1i})\right|}$$ (19)

$$U_{2}(s_{2})=\sqrt[{n}]{\left|\prod_{i}^{n}U_{2}(s_{2i})\right|}$$ (20)

After computing the subsystem order, the composite system C(t) degree has been computed using Eq (21)

$$C(t)=\sqrt[{\theta }]{\left|\prod_{j}^{n}\left[u_{j}^{t}(e_{j})-u_{j}^{0}(e_{j})\right]\right|}$$ (21)

Here, the computed *C*(*t*) value has (-1,1); $\theta =\frac{min[u_{j}^{t}(e_{j})-u_{j}^{0}(e_{j})\neq 0]}{\left|min[u_{j}^{t}(e_{j})-u_{j}^{0}(e_{j})\neq 0]\right|}$. The computed degree of value determines subsystem constituents. According to the subsystem degree of value, the high coordination between digital logistics and the digital economy is determined effectively. Likewise, the different economic models are incorporated to identify the association between logistics and economic development. The analysis clearly states that statistical models and big data technology are incorporated into the logistic environment to improve overall business economic growth. The logistics environment utilizes the data aggregation process that rapidly maximizes the regional economy. However, the existing techniques require additional effort while analyzing spatial measurement in the logistics industry. In addition, transportation in logistics consumes high computation costs which cause to affect the industry’s economic growth. Therefore, this study uses Dynamic Stochastic Equilibrium with Statistical Analysis Modelling (DSE-SAM) to improve the industrial economy by considering the logistics industry.

## 3. Materials and methods

Digital transformations are driven by the emergence of new business models that use digital technology to build solutions and value-added services while lowering costs. Companies will attempt to incorporate more value-added processes as they recognize that the more efficient, they become due to better logistics management (i.e., adopting digitalization), the more value-added activities they want to integrate. As a result, operational and organizational structure modifications are starting to be based on how well assets are being used. In certain cases, companies understand the value of providing excellent customer service and want to gain an advantage in the marketplace by improving their responsiveness to customers’ needs. This section discusses the materials used to examine the relationship and correlation between logistics and economic development. According to various researchers, the Yangtze River Economic Belt region [23] is utilized in this work. The data is collected from 2009 to 219; here, 2022 sustainability is utilized in 24 provincial-level regions. Each region has specific indicators that help to compute the region’s economic level. The indicators are determined from the statistical yearbook (2009 to 2019), statistical bulletins of social & economic development, the Yangtze River economic belt book (2009 to 2019), and the China environmental yearbook (2009 to 2019). These books extract various indicators for resources, such as logistics industries, population urbanization, and ecological environment. The extracted indicators are described in Table 1.

**Table 1:** Indicators utilized in the region’s economic level analysis.

System	Primary Indicators	Secondary Indicators
Logistic	Infrastructure	Mileage of railway Mileage of road Outlets of postels Civilian cargo ownership
	Scale of development	
Urbanization	Population	Total population (%) and population density
	Economic	Per capita GDP, GDP of the secondary sector, GDP of the tertiary sector, and urban resident disposable income
	Social	Vehicle count of public transport, public toilet counts, number of beds in healthcare, number of students in the education environment.
	Spatial	Urban road information and proportion of the urban area.
Ecological	Status	Forest area, green region, green space occupied by the person
	Pressure	Water discharge from industries, emission of sulfur dioxide, and smoke emission

Table 1 based collected indicators is collected from the Yangtze River Economic Belt region [24]. The Yangtze River Economic Belt dates back to the 1980s, when it was suggested as an extension of the Yangtze River Industrial Concentration Zone. The region has 11 cities: Shanghai, Anhui, Zhejiang, Jiangsu, Hunan, Guizhou, Yunnan, Sichuan, Hubei, Jiangxi, and Chongqing. The Yangtze River economic belt idea, which bases regional growth on the world’s third biggest river basin unit, is important to the establishment of an ecologically sustainable society. About 30 percent of China’s GDP is produced in the Yangtze River Basin, making it a key contributor to the country’s fast economic development. With a population of 600 million and a GDP contribution of 45 percent, the region accounts for 21.4% of China’s total geographical area and generates $2.0523 trillion annually.The Yangtze River Economic Belt comprises an upper, middle, and bottom segment [25].

The Yangtze River Economic Belt has negative coefficients for the entire basin and the upstream sector in terms of foreign commerce, whereas the downstream and the middle regions have positive coefficients. Except for the upstream area model, international trade’s impact was statistically significant at the 1% level in all other regions’ models. Overall, the results suggest that increasing the level of commerce has a major negative impact on the development of economic rate in the region, contrary to theoretical assumptions. It is important to shift the focus from the conventional quantitative effect of international commerce to the quality effect in order to boost trade and encourage high-quality economic growth along the Yangtze River Economic Belt. While there are clear variations between the upstream, midstream, and downstream, regionally speaking, the coefficients of trade in the relevant models for these three regions are 2.043, 1.869, and 5.252 [26].

The Yangtze River Economic Belt Region consists of 11 cities. These cities are composed of logistics, ecology, and urbanization. These systems are highly utilized to identify the relationship and correlation between the logistics process and the national economy. These systems are analyzed continuously according to different indicators to calculate the evaluation index for logistics, ecology, and urbanization environment. Initially, the primary indicators for logistics, ecology, and urbanization system are detected to understand the infrastructure [27]. The identified indicators help to improve China’s industrial development. Second, the China logistic industries are investigated according to infrastructure and development. Third, new urbanization is analyzed in terms of spatial, social, economic, and population urbanization. These four perspectives are further analyzed to get the secondary indicators such as population, Gross Development Product (GDP), vehicle count, and urbanization transport information [28]. The last system is the ecology environment which is examined in terms of development and status level. From the primary indicators, ecological environment-related secondary indicators such as government attention, pollution control, and green region indicators are extracted. This procedure is followed continuously to extract the different indicators according to the social, spatial, accessibility, and scientific principles [8]. Finally, the derived indicators are processed by statistical procedure to extract the weight index value used to determine the connection between logistics and the province’s economic development.

### 3.1 Dynamic Stochastic Equilibrium with Statistical Analysis Modelling (DSE-SAM)

**Fig 1:**
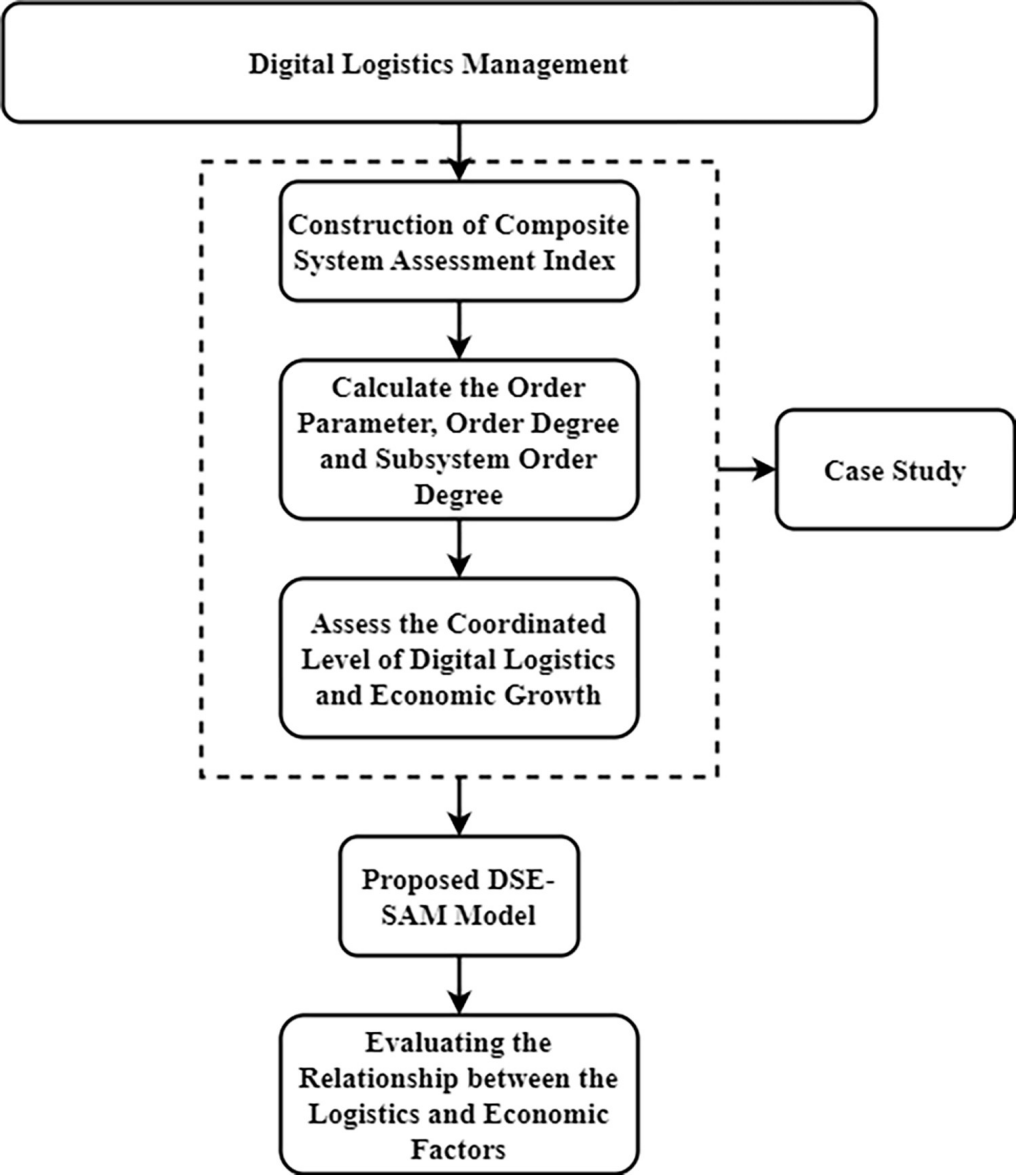
Proposed DSE-SAM model.

Fig 1 shows the proposed DSE-SAM model. The statistical analysis modeling approach collects and processes the Yangtze River Economic Belt Region-related information. The modeling approach predicts the link between logistics and the economy for positive and negative indicators. According to various research studies, the logistic development evaluation index is difficult to manage because of the high computation cost. In addition, most researchers use different factors to investigate and explore the industry development toward economic growth. However, the existing modeling techniques have high computation complexity to create the unique evaluation index because existing techniques deal with the index values for primary and secondary indicators. This study concentrates on the Yangtze River Economic region city’s logistic industry development. Different indicators are used to compute the evaluation index values during the analysis. Table 1 shows three systems, such as logistics, ecology, and urbanization, that are evaluated frequently to derive the eight primary and 22 secondary indicators. These indicators are extracted depending on the infrastructure, industrial scale, industrial support, and human resources. The Yangtze River region information is analyzed to form the judgment matrix, which is defined using Eq (22)

$$JM={\left(x_{ij}^{t}\right)}_{mT*n};\;1\leq i\leq m,1\leq j\leq n,1\leq t\leq T$$ (22)

In Eq (22), JM is denoted as the judgment matrix, computed from the collected information from region m using evaluation indices n at a specific time T. After forming the JM, the normalization process is performed to minimize the missing value involvement. During this process, Eq (1) is utilized for normalizing the positive indicator, and Eq (2) is utilized to perform the negative indicator normalization. For the logistic industries, almost every second-level indicators are positive; therefore, Eq 1 is utilized to normalize the indicators in the logistic industry. Afterward, each indicator’s entropy is calculated to identify the weight value. The entropy value is estimated using Eq (23)

$$e_{j}=\frac{1}{\ln\;mT}\sum_{t=1}^{T}\sum_{i=1}^{m}\left[\frac{{\left(x_{ij}^{t}\right)}^{\prime }}{\sum_{t=1}^{T}\sum_{i=1}^{m}{\left(x_{ij}^{t}\right)}^{\prime }}\ln\frac{{\left(x_{ij}^{t}\right)}^{\prime }}{\sum_{t=1}^{T}\sum_{i=1}^{m}{\left(x_{ij}^{t}\right)}^{\prime }}\right]$$ (23)

From Eq (23), the coefficient variability *g*_*i*_ is computed as 1−*e*_*j*_ which helps to identify the weight index value *w*_*j*_ which is estimated according to Eq (5). These computed factors are applied to estimate the logistics industry’s comprehensive index value (Eq 24).


$$F_{i}^{t}=\sum_{j=1}^{n}w_{j}{\left(x_{ij}^{t}\right)}^{\prime }$$ (24)

The computed $F_{i}^{t}$ directly shows the importance of the primary and secondary indicators while analyzing the logistics and provincial economic development association. The region’s economic development is identified based on the region’s competitiveness and comprehensive strength. Generally, the GDP/per capita value is utilized to calculate the regional economic growth. In addition, the core explanatory and regional variables highly influence regional economic development. The industries maintain the variable lists that include the government function, labor input, outside degree open, urbanization, and capital investment. This list identifies the relationship between the industry’s logistics and economy level. During the analysis, the Cobb-Douglas production function is utilized to explore the impact of logistic development on regional economic development. The production function is defined as *ED* = *F*(*lde*, *lp*, *gf*, *ci*, *do*, *u*). Here ED-is defined as the economic development that is computed from the per capita GDP value, *lde* -Logistic industry entropy, which measures the comprehensive development, *lp*-number of employees in the industries (labor input), *gf* is defined as government functions on education, science, and technology, *ci* is represented as capital input, *do* is mentioned as the degree of openness in outside, and *u* is signified as urbanization. The *ED* is further defined according to the regression coefficient linearization model, which is represented in Eq (25)

$$\ln\;ED=\beta_{0}+\beta_{1}\;\ln\;lde+\beta_{2}\;\ln\;lp+\beta_{3}\;\ln\;gf+\beta_{4}\;\ln\;ci+\beta_{5}\;\ln\;do+\beta_{6}\;\ln\;u+\varepsilon$$ (25)

In Eq (25), error terms are defined as *ε*, belonging to normal distribution. As said, the logistic environment highly influences regional economic factors because it has spatial connections-related characteristics. Therefore, spatial autocorrelation is computed to identify the relationship between each factor which is defined using Eq (26)

$$I_{i}=\frac{\left(x_{i}-\bar{x}\right)}{s^{2}}\sum_{j=1}^{n}w_{ij}(x_{j}-\bar{x})$$ (26)

In Eq (26), s is denoted as the variance value and $\bar{x}$ is mentioned as the mean value. If *I*_*i*_ greater than zero, then the logistic industry and regional economic growth have high or strongly correlated (positive correlation). If the *I*_*i*_<0, then both factors, such as logistics and economics, correlate negatively. Suppose, *I*_*i*_ = 0, logistic and economy has the no-spatial clustering in the area. In addition, dynamic stochastic equilibrium modeling is applied to identifying and forecasting the economic factor using economic policies. Here, the regional area’s related time series data is utilized to identify the economic growth factor in the logistics industry. The regional central bank and government utilize this equilibrium model to investigate economic policies and factors. The created policies cover the monetary policies, supply, and demand, which helps identify the strong relationship between logistics and economic factors. Then the equilibrium model is defined using Eq (27)

$${ip}_{t}=\alpha E(r_{t+1}+e_{t})$$ (27)

In Eq (27), the economic output *ip*_*t*_ for the region, the indicator is obtained from the interest rate (r), latent factor influencing the interest rate m, and latent factor influencing the production e. Here, *r*_*t*+1_ is estimated using *βip*_*t*_+*m*_*t*_ and evaluating factor is defined using Eq (28)

$$e_{t+1}=\theta_{1}e_{t}+\theta_{2}e_{t-1}+u_{t+1}$$ (28)

According to the above computation, economic growth has been predicted in the region. Then it helps predict the strong association between logistics and provincial economic growth.

## 4. Results and discussions

This paper uses Dynamic Stochastic Equilibrium with Statistical Analysis Modelling (DSE-SAM) to analyze digital logistics’ impact on economic growth. The data are selected from the dataset of to promote the coordinated development of the digital economy and logistics industry.https://www.kaggle.com/datasets/theworldbank/data-resources-for-structural-economic-analysis [29]. A standardised collection of metadata for more than 60 worldwide databases on the global economic structure, including data characteristics and access information. Consistent metadata comprising the technical aspects of the data and access information is compiled for approximately 60 worldwide datasets on the structure of the global economy. Production and value added by industry, labour force, social and demographic statistics, productivity, and indicators of economical endowments are all included in this compilation. According to data scientificity and availability principles, index systems are built from the growth level of the digital economy and logistics. The Z-score technique deals with the data’s lack of dimensions. Therefore, the data-collection progression primarily gathers appropriate data reproducing the growth level of digital logistics and the economy. Next, the actual data are processed utilizing Z-scores, standards and dimensionless techniques. After that, data modeling is utilized to construct synergy models of composite systems of the digital logistics and provincial economy. The national logistics policy is meant to make the country’s economy’s growth easier by lowering logistics costs and making domestic goods more competitive on the world market. Information technology helps partner firms coordinate logistics activities, establish electronic connections, and digitize logistics to enhance logistical coordination. Furthermore, innovative uses of technologies in various operations, such as data analytics, the internet of things, and the cloud, are disrupting existing business models. As a result, many parties in the supply chain are quickening at diverse speeds and challenging expectations and developments in the digital transformation economy. This section analyzes the comprehensive results analysis of different systems, such as logistics, urbanization, and ecology in the Yangtze River region. First, the Yangtze region information is explored using the entropy model that predicts every indicator’s importance and probability value. Then, in line with the entropy value (expression 22), the total index value is computed, which utilizes the indicator weight and coefficient variability value. Finally, the obtained index value for various cities in the Yangtze River region is illustrated in Tables 2–4.

**Table 2:** Logistic industry comprehensive index value for various regions.

Cities	2009	2010	2011	2012	2013	2014	2015	2016	2017	2018	2019
Zhejiang	0.9266	0.9307	0.9342	0.9398	0.9313	0.9357	0.9219	0.9284	0.9274	0.9207	0.928
Jiangsu	0.9268	0.9389	0.9322	0.9268	0.9426	0.9432	0.944	0.9482	0.9436	0.9203	0.9428
Anhui	0.683	0.6311	0.7081	0.6359	0.726	0.6202	0.756	0.7581	0.6439	0.7239	0.7277
Shanghai	0.7098	0.6485	0.6777	0.6533	0.6803	0.6957	0.6676	0.6624	0.6541	0.7126	0.6584
Hunan	0.4382	0.4065	0.4348	0.5471	0.4038	0.3936	0.5456	0.4055	0.4358	0.5172	0.3555
Guizhou	0.3857	0.4676	0.4493	0.448	0.4908	0.4712	0.4581	0.5062	0.3407	0.5152	0.4207
Yunnan	0.6249	0.6156	0.5979	0.6347	0.6455	0.6492	0.5455	0.5499	0.5858	0.5623	0.5374
Sichuan	0.6326	0.5679	0.681	0.689	0.6665	0.6199	0.6173	0.7219	0.6383	0.7157	0.7042
Hubei	0.3863	0.3477	0.4226	0.3589	0.4569	0.5071	0.3813	0.4834	0.3735	0.4495	0.4984
Jiangxi	0.5126	0.4426	0.5172	0.5082	0.4688	0.4306	0.4747	0.424	0.5226	0.377	0.4403
Chongqing	0.4238	0.3412	0.4103	0.3672	0.5472	0.3895	0.537	0.3986	0.392	0.4099	0.4939

**Table 3:** Urbanization system comprehensive index value for various regions.

Cities	2009	2010	2011	2012	2013	2014	2015	2016	2017	2018	2019
Zhejiang	0.9345	0.934	0.9328	0.9268	0.924	0.9388	0.9256	0.9317	0.9392	0.9332	0.9282
Jiangsu	0.9479	0.9294	0.9315	0.927	0.928	0.9329	0.9282	0.9465	0.9297	0.9395	0.9223
Anhui	0.6524	0.6222	0.6293	0.6931	0.6592	0.7279	0.6245	0.6764	0.7235	0.6989	0.6624
Shanghai	0.6494	0.7357	0.6488	0.68	0.6526	0.654	0.7474	0.7181	0.6477	0.6874	0.7398
Hunan	0.54	0.5253	0.503	0.3668	0.5239	0.4721	0.4664	0.4705	0.4775	0.3968	0.3694
Guizhou	0.4959	0.5044	0.5452	0.5212	0.3417	0.4846	0.4236	0.5349	0.5296	0.5287	0.3545
Yunnan	0.5795	0.6294	0.5545	0.5623	0.543	0.5509	0.6238	0.5312	0.6342	0.643	0.5286
Sichuan	0.6973	0.57	0.6725	0.5985	0.7213	0.6176	0.5551	0.5894	0.7169	0.6416	0.708
Hubei	0.4459	0.3886	0.4953	0.4994	0.4236	0.3431	0.4594	0.3566	0.5351	0.4809	0.481
Jiangxi	0.5172	0.4208	0.4293	0.4105	0.457	0.4057	0.4598	0.4958	0.5404	0.42	0.4316
Chongqing	0.538	0.373	0.364	0.5254	0.5015	0.5311	0.5077	0.5192	0.5168	0.4097	0.4669

**Table 4:** Ecological systems comprehensive index value for various regions.

Cities	2009	2010	2011	2012	2013	2014	2015	2016	2017	2018	2019
Zhejiang	0.9253	0.922	0.9341	0.9282	0.9384	0.9266	0.9323	0.9311	0.9235	0.932	0.9267
Jiangsu	0.9467	0.9434	0.9247	0.9482	0.9243	0.9238	0.9203	0.931	0.9383	0.9468	0.9228
Anhui	0.7112	0.6954	0.7498	0.699	0.697	0.7058	0.705	0.7246	0.7187	0.7576	0.6716
Shanghai	0.6512	0.6818	0.6707	0.6471	0.747	0.6658	0.723	0.7419	0.6629	0.6954	0.6824
Hunan	0.3869	0.55	0.5193	0.4315	0.4156	0.3703	0.4187	0.4134	0.5297	0.5407	0.4708
Guizhou	0.3462	0.5059	0.4242	0.5422	0.3417	0.4492	0.4551	0.5206	0.3801	0.5469	0.4938
Yunnan	0.5322	0.6437	0.5384	0.5634	0.5482	0.5626	0.6383	0.5689	0.6056	0.5367	0.5769
Sichuan	0.6245	0.594	0.6278	0.5492	0.5448	0.5558	0.6888	0.5467	0.6789	0.6076	0.6781
Hubei	0.5355	0.4936	0.4617	0.412	0.4326	0.5225	0.3416	0.4052	0.521	0.5458	0.3779
Jiangxi	0.3649	0.5257	0.3448	0.4865	0.4666	0.4094	0.4666	0.3717	0.3664	0.3778	0.3542
Chongqing	0.4986	0.4457	0.5235	0.4867	0.4778	0.4711	0.5287	0.5452	0.4254	0.5231	0.5056

Tables 2–4 represent the comprehensive index value for the three systems: logistics, urbanization, and ecological. Here, the entropy approach (Eq 1) is utilized with the weight factor to compute the comprehensive index value (Eq 23) for every indicator in the region. The analysis uses information from 2009 to 2019 in the Yangtze River economic region. The computed comprehensive index value was used to identify the relationship between logistics and economic growth at the development level. The table clearly states that the economic growth values are gradually increasing horizontally, which means the economic growth is increased yearly due to the proper maintenance of the logistic feature. The analysis of China’s province, Zhejiang, and Jiangsu regions have gradual development in their economic activities because of effective railways, highways, and transportation. In addition, effective logistic transportation enhances the Zhejiang province’s shopping and other activities, increasing industrial economic growth. The other regions, like Guizhou and Yunnan, are located in remote areas; therefore, they have minimum transportation facilities influencing the logistics process. The minimum logistic leads to reduce the region’s development and economy. During the analysis, the system uses the 22 indicators that help analyze the sustainability of the industry development on various systems. Table values 2, 3, and 4 clearly state that the Yangtze River region has the better improvement on three streams: logistics, ecology, and urbanization.

Then, the Statistical Analysis Modelling is applied to the region’s data to evaluate the spatial relationship between the region’s economic growth and respective logistics development. The economic development is analyzed with the help of the linear regression coefficient model defined in Eq (24). The coefficient model gives the high degree values in the Yangtze River Economic region. From 2009 to 2019, each region gradually increased its participation. In 2009, the region had economic coordination values between 0.45 and 0.67, which increased in 2019 from 0.76 to 0.89. Likewise, the link between logistics and the economy has been analyzed in the urbanization system and ecological environment. This environment utilizes different factors such as the number of populations, per capita GDP, population density, etc. These factors are highly influencing the logistic process to maximize economic development. In addition, the relation between the indicators is examined for various statuses and development in the ecological system. The effective utilization of leadership indicators protects green regions and green development. The green regions also help to maximize the overall GDP. In China, The Jiangsu, Shanghai, and Zhejiang regions have gradually improved this production from 2009 to 2019. Zhejiang links the logistics and the region’s economic development among the various provinces. Similarly, other provinces meet the primary link between the logistic and economic factors for primary and secondary indicators. However, Guizhou requires additional effort to maximize its association with ecological systems.

The influence of logistics on the region’s economic development has been evaluated in the Yangtze River Economic region. The economic belt has 11 provinces, in which Eq (24) identifies the coordination of economic development. The spatial analysis of these cities [20], which is analyzed with the help of the ArcGIS software. Spatial Coordination Analysis of Logistics and the Economy in the Yangtze River Belt Region in 2009, 2014, and 2019 was empirically analysed using the coupling coordination degree model and the spatial auto correlation analysis model, which shed light on the coordination of the economy in the Yangtze River Economic Belt and its relationship of mutual influence.

- 2009 Spatial Coordination of logistic and Economy
- 2014 Spatial Coordination of logistic and Economy
- **2019 Spatial Coordination of Logistics and Economy**

The spatial coordination analysis of logistics and economy in the Yangtze River belt region. Here, the province has spatial distribution and coordination between the logistics and economy. In addition, Zhejiang, Shanghai, and Jiangsu have effective coordination among the different cities. China’s firms in the Yangtze River Economic Belt were studied in terms of their spatial coordination, logistics, and economic features. In light of these findings, the research conducts a Spatial Coordination Analysis of Logistics and Economy in the Yangtze River Belt Region in 2009, 2014, and 2019. Based on the coupling coordination model to examine the coupling coordination connection between logistics and economic systems, the relative development degree is utilised to indicate the comparative development state of the logistics industry and the economy. Facilitating coordinated growth in logistics and the economy is essential to fostering high-quality economic expansion, which in turn improves people’s standard of living and sense of well-being.

Ecological and economic growth were most effectively coordinated in Zhejiang Province. The most concentrated region of high co-ordination degree in the whole economic belt was found at the border of Zhejiang, Anhui, and Jiangxi Provinces. Many counties (districts) in central and western Sichuan Province, as well as the northern region of Jiangsu Province, have a poor co-ordination degree. Most districts and counties of Jiangxi, Hunan, and Hubei Provinces have a co-ordination degree more than 0.49, making them among the most coordinated areas in China.

Coordination between couplings was poor in the Sichuan Basin. The poor level of coordination may be traced back to the prevalence of moderate disorder in several central counties (districts), including Jinyang City, Jintang County, Neijiang City District, and Ziyang City District. Coupling coordination had advanced swiftly over much of Yunnan Province, with counties in the Chuxiong Yi Autonomous Region Notable was the sustained improvement in Suzhou City, Jiangsu Province’s coupling coordination level from 2009 to 2019 in light of the city’s already high degree of co-ordination to begin with. In 2019, the variability of Zhejiang Province’s CCD continued to decrease and extended to neighbouring provinces. Zhejiang Province had the highest concentration of barely coordinated, balanced, and high-quality ecological-economic development. This coordination is computed using Eq 24, and the obtained outcomes are illustrated in Table 5.

**Table 5:** Spatial coordination analysis of logistics and economy.

Cities	2019		2014		2019	
	Value	Degree	Value	Degree	Value	Degree
Zhejiang	0.67	PC	0.546	BC	0.83	GC
Jiangsu	0.65	PC	0.572	BC	0.756	IC
Anhui	0.587	BC	0.462	NC	0.684	PC
Shanghai	0.539	BC	0.689	PC	0.548	BC
Hunan	0.498	NC	0.542	BC	0.623	PC
Guizhou	0.432	NC	0.489	NC	0.587	BC
Yunnan	0.487	NC	0.321	MC	0.542	BC
Sichuan	0.428	MC	0.319	MC	0.518	BC
Hubei	0.576	BC	0.472	NC	0.539	BC
Jiangxi	0.589	BC	0.423	NC	0.634	PC
Chongqing	0.498	NC	0.418	NC	0.52	BC

Note—PC-Primary coordination, NC-Near Coordination, BC-Basic Coordination, GC-Great Coordination, IC-Intermediate Coordination.

Table 5 clearly states that the Zhejiang region highly correlates with the logistic economy. The Yangtze River Economic belt region successfully utilizes the resources, talents, and technologies which cause the identification of the relationship between logistics and economy in different systems. Few regions have basic and near coordination; however, they are highly incorporated to improve their economic growth by concentrating the region’s logistic information. Overall, the river region cities are gradually maximizing their contribution to the region’s economic growth with minimum difficulties. However, the system’s efficiency is evaluated with different economic models to analyze how effectively the introduced system attains the relationship between logistics and the economy. The efficiency of the introduced Dynamic Stochastic Equilibrium with Statistical Analysis Modelling (DSE-SAM) is compared with the other economic models, such as Coupling Coordination Degree Model (CCDM), Spatial Durbin Model (SDM), and Collaborative Degree Model (CDM). Initially, the correlation between digital logistics and economy is computed using Eq 25, and the obtained results are compared with the other model’s correlation which is estimated using Eq (8), Eq (15), and Eq (20). The obtained correlation results for various cities are illustrated in Table 6.

**Table 6:** Correlation analysis of various economic models.

Cities	DSE-SAM	CCDM	SED	CDM
Shanghai	0.9258	0.7205	0.6847	0.6442
Anhui	0.9342	0.755	0.6864	0.6491
Zhejiang	0.9394	0.7519	0.6503	0.6411
Jiangsu	0.9397	0.7353	0.63	0.6447
Hunan	0.9235	0.741	0.6301	0.6439
Guizhou	0.9334	0.7321	0.6279	0.6464
Yunnan	0.9298	0.7244	0.6607	0.643
Sichuan	0.9318	0.7269	0.6729	0.6401
Hubei	0.939	0.7327	0.6854	0.6465
Jiangxi	0.9208	0.7592	0.6614	0.6459
Chongqing	0.9223	0.7596	0.6214	0.646

Table 6 clearly shows that the introduced Dynamic Stochastic Equilibrium with Statistical Analysis Modelling (DSE-SAM) attains high correlation values compared to another economic model. Here, the financial policies are equilibrium identified which helps to predict the logistic impacts on the region’s economic factor. In addition, the method uses the 22 secondary indicators that are highly helpful in predicting the positive and negative impact on the industry’s economic growth. From the analysis, Sichuan, Jiangsu, and Zhejiang cities have a high correlation because of successfully generating the judgment matrix for every piece of information. In addition, the normalization process helps to remove the missing value and inappropriate values. This process maximizes the correlation between the primary and secondary factors in every system, like logistics, urbanization, and ecological systems. In addition, spatial autocorrelation analysis is performed to identify the strong relationship between logistics and the region’s economy.

Table 7 refers to the spatial correlation analysis of the region’s economic growth and logistic industries. Here the correlation is performed according to the coordination degree defined in the Eq (25). The obtained results of different regions in various years are examined in each city having the spatial correlation between each primary and secondary indicator. Thus, each region obtains high correlation values compared to the other economic model, which directly shows that every single step of logistic industries leads to maximizing the region’s economic growth.

**Table 7:** Spatial autocorrelation analysis of various economic models.

Cities	DSE-SAM	CCDM	SED	CDM
Shanghai	0.9326	0.7466	0.6439	0.6427
Anhui	0.9259	0.7472	0.6345	0.643
Zhejiang	0.9264	0.7247	0.6602	0.6452
Jiangsu	0.9393	0.7595	0.6551	0.6482
Hunan	0.9386	0.7595	0.6435	0.6433
Guizhou	0.9246	0.73	0.6898	0.6431
Yunnan	0.9288	0.7557	0.6667	0.6494
Sichuan	0.9291	0.7303	0.6515	0.6421
Hubei	0.9232	0.7592	0.6674	0.6452
Jiangxi	0.9369	0.7588	0.6387	0.6433
Chongqing	0.9236	0.73	0.6887	0.6484

## 5. Conclusion

Thus, the paper analyzes the Dynamic Stochastic Equilibrium with Statistical Analysis Modelling (DSE-SAM) based impact analysis of logistic and regional economic growth. This study uses the Yangtze River Economic Belt Region is information is utilized to evaluate the relationship between the logistics and economic factors. The collected information is processed to form the judgment matrix, which is more useful for identifying the relationship between the indicators. From the judgment matrix, entropy values are obtained from the normalized features. Then the correlation between the indicators is computed along with the weight matrix. This process predicted the strong relationship between logistics and economic activities on logistics, urbanization, and the ecological environment. The created system is evaluated in terms of development-based, spatial, and autocorrelation analysis. According to the various analyses, the DSE-SAM approach strongly correlates with logistics and the economy. The obtained results have a high correlation compared to the other economic model. Future studies will be further enhanced by applying optimized correlation procedures on primary and secondary indicators to improve the relationship analysis. The limitation of this study outcome is the difficulty of standardizing an information technology solution for developing and implementing self-organizing digital logistics since each company has specific business models and already has one or more customized information technology systems. Furthermore, more elements impact digital logistics and the economy due to societal growth; the index system for the composite system as a whole has to be improved in future research.

## Supporting information

S1 File (DOCX)
