# Supplementary material for: The impact of digital logistics under the big environment of economy
Source: PLoS One. 2023 Apr 6;18(4):e0283613. doi: 10.1371/journal.pone.0283613 (PMC10079085; doi:10.1371/journal.pone.0283613)
Supplement: S1 File — (DOCX) [file pone.0283613.s001.docx]

https://www.kaggle.com/datasets/theworldbank/data-resources-for-structural-economic-analysis.

A standardized collection of metadata for more than 60 worldwide databases on the global economic structure, including data characteristics and access information. Consistent metadata comprising the technical aspects of the data and access information is compiled for approximately 60 worldwide datasets on the structure of the global economy. Production and value added by industry, labour force, social and demographic statistics, productivity, and indicators of economical endowments are all included in this compilation
